# Paper modified with ZnO nanorods – antimicrobial studies

**DOI:** 10.3762/bjnano.3.78

**Published:** 2012-10-11

**Authors:** Mayuree Jaisai, Sunandan Baruah, Joydeep Dutta

**Affiliations:** 1 Center of Excellence in Nanotechnology, Asian Institute of Technology, Klong Luang, Pathumthani 12120, Thailand; 2 Assam Don Bosco University, Azara, Guwahati 781017, India; 3 Chair in Nanotechnology, Water Research Center, Sultan Qaboos University, PO Box 17, Postal Code 123, Al Khoud, Oman

**Keywords:** antimicrobial, nanorod, paper, photocatalysis, zinc oxide

## Abstract

Paper with antimicrobial properties was developed through in situ growth of ZnO nanorods. The targeted application for this type of paper is in health centers as wallpaper, writing paper, facemasks, tissue paper, etc. The paper was tested on three model microbes, Gram-positive bacteria *Staphylococcus aureus,* Gram-negative bacteria *Escherichia coli* and common airborne fungus *Aspergillus niger*. No viable bacterial colonies or fungal spores could be detected in the areas surrounding test samples of the antimicrobial paper. Gram-negative bacteria *Escherichia coli* were found to be inhibited in an area that is 239% and 163% the area of the paper sample under different room lighting conditions, i.e., halogen and fluorescent lamp illumination, respectively. For Gram-positive bacteria *Staphylococcus aureus* the zones of inhibition surrounding the paper samples are 102% and 70%, and for *Aspergillus niger*, 224% and 183% of the sample area, under similar lighting conditions.

## Introduction

Deterioration of library materials due to fungal growth is a worldwide problem and a cause of extensive damage to precious books and manuscripts [1–2]. In relation to this, documents in hospitals and research centers are carriers of infectious agents, such as disease-causing bacteria and viruses. Extreme care needs to be taken especially by people working in such organizations while handling documents. In addition, facemasks are not always capable of providing protection from the transmission of infectious diseases as they can themselves be carriers of harmful microorganisms [3].

The most common fungi that grow on paper are the air-borne *Aspergillus* and *Penicillium* [1]. Normally, fungal infections in books are treated by using chemical methods. Libraries need to be maintained at a temperature and relative humidity that are not conducive to fungal growth [1]. Paper with antimicrobial properties could be an answer to the problems faced by libraries and health centers. Silver (Ag) nanoparticles embedded into a paper matrix have been reported as exhibiting antibacterial properties [4]. Wallpaper prepared by using zinc oxide nanoparticle (~20 nm) coatings has been reported to render antibacterial surfaces that inhibit growth of bacteria such as *Escherichia coli* (*E. coli*) [5]. An increase in cellular internalization of ZnO nanoparticles has also been observed by Appierot et al. [6] in a study of their antibacterial effect on *E. coli* and *S. aureus*.

This work reports on an antimicrobial paper containing zinc oxide (ZnO) nanorods grown by a hydrothermal process, and which can be used for various applications, such as facemasks, tissues, wallpapers and writing paper. The antimicrobial effect of the ZnO nanorods grown on the paper matrix results from the slow seepage of Zn^2+^ ions assisted by atmospheric humidity or through the injection of free electrons resulting from photocatalysis [7–8]. Cellulose fibers used for papermaking are hygroscopic in nature [9–10] and this property was used to our advantage when developing the antimicrobial paper. The adsorbed moisture can be utilized for the production of hydroxyl radicals (·OH) and/or hydrogen peroxide (H_2_O_2_) through photocatalysis.

Both ·OH and H_2_O_2_ are harmful to the cells of living organisms and are the major contributors to antibacterial activity [11–13]. ZnO nanoparticles are reported to have significant antifungal properties against *B. cinerea* and *P. expansum*, and the inhibitory effects were found to increase with an increase in the concentration of the nanoparticles [14]. Other metal oxides, such as iron oxide, also exhibit antibacterial and antifungal properties, as have been reported by Prucek et al*.* [15].

In a photocatalysis process, electron–hole pairs are generated through photonic excitation of wide-band-gap metal-oxide semiconductors, such as ZnO, titanium dioxide (TiO_2_), etc. The photogenerated free carriers allow efficient mineralization of toxic organic compounds [16] and hazardous inorganic materials [17], and microbial disinfection [18] through the creation of a hydroxyl radical (OH·), which acts as a strong oxidizing agent [17]. The reactions initiated by photogenerated electrons, leading to the formation of hydroxyl radicals and hydrogen peroxide, are summarized as follows [19]:


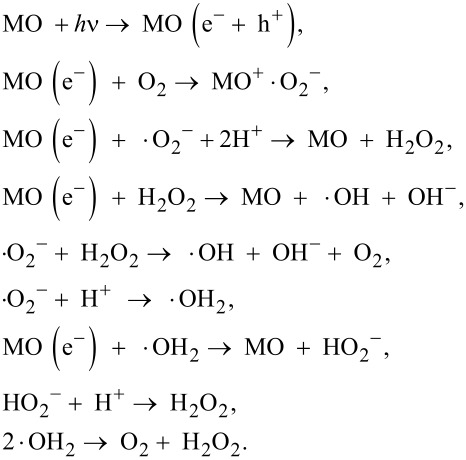


where MO stands for metal-oxide photocatalyst, such as TiO_2_, ZnO, etc., and the reaction products and intermediates are superoxide anions (·O_2_^−^), hydrogen peroxide (H_2_O_2_), hydroxyl radicals (·OH), hydrogendioxide anion (HO_2_^−^), and hydroperoxy radicals (·HO_2_).

Surface area and surface defects play an important role in the photocatalytic activity of metal-oxide nanostructures. One-dimensional nanostructures such as nanorods offer large surface-to-volume ratios. Hydrothermally grown ZnO nanorods possess inherent defects in the form of oxygen vacancies and zinc interstitials, which shift its optical absorption from the ultraviolet to the visible region [20]. We previously reported the visible-light photocatalytic degradation of organic dyes using similar paper embedded with ZnO nanorods [21]. In this work we report the antimicrobial activities of paper functionalized by in situ growth of ZnO nanorods through a hydrothermal process.

## Results and Discussion

**Figure 1:**
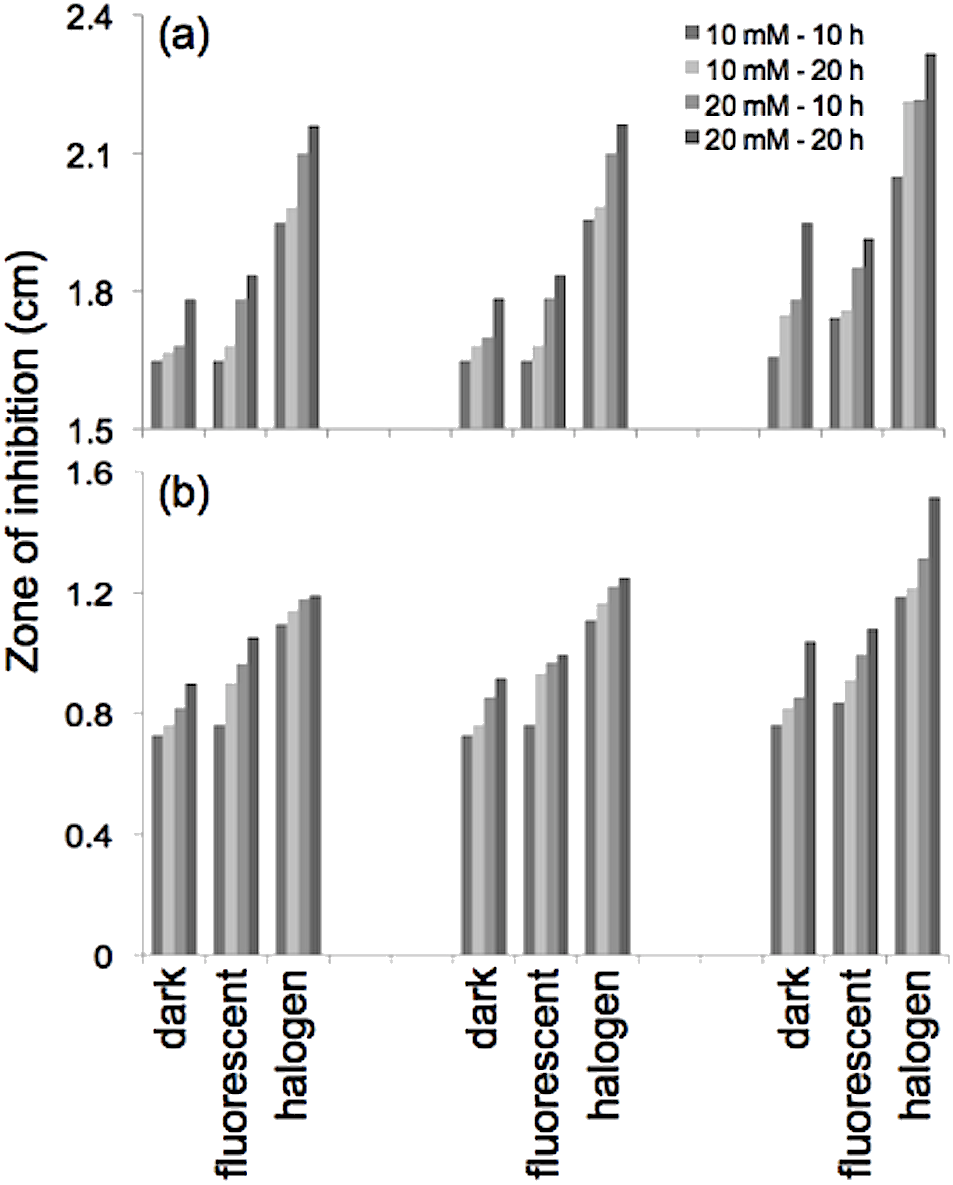
Increase in zone of inhibition for *E. coli* and *S. aureus* with increasing incubation time under dark conditions and upon light illumination in the presence of antimicrobial paper. No zone of inhibition was observed for the control sample without ZnO nanorods. The antimicrobial paper was more effective in immobilizing Gram-negative *E. coli* as they have a thinner cell wall compared to Gram-positive *S. aureus*.

Studies on the photocatalytic immobilization of *E. coli* and *S. aureus* reveal that the antimicrobial paper is more effective against *E. coli*, as was also reported in our previous publication [21] and other photocatalysis studies [22]. This can be attributed to the thinner cell wall of Gram-negative bacteria compared to the Gram-positive type. Figure 1 shows the measure of zones of inhibition (edge of the square inhibition zone in centimeters) around the paper squares of side 1.5 cm for different samples with ZnO nanorods. The antimicrobial activity in the dark is due to the slow release of Zn^2+^ ions arising from partial dissolution of ZnO in the moist environment leading to the rupture of the bacterial cell wall [23].

*S. aureus*, being a Gram-positive bacterium, has a thicker cell wall [24], and consequently its immobilization by using the ZnO-coated antimicrobial paper is comparatively lower than that of *E. coli*. The highest antimicrobial activity was observed in paper samples coated with ZnO nanorods grown at a concentration of 20 mM for 20 h, which is attributed to the higher effective surface coverage of ZnO nanorods allowing more bacterial cells to come into contact with the ZnO surface.

**Figure 2:**
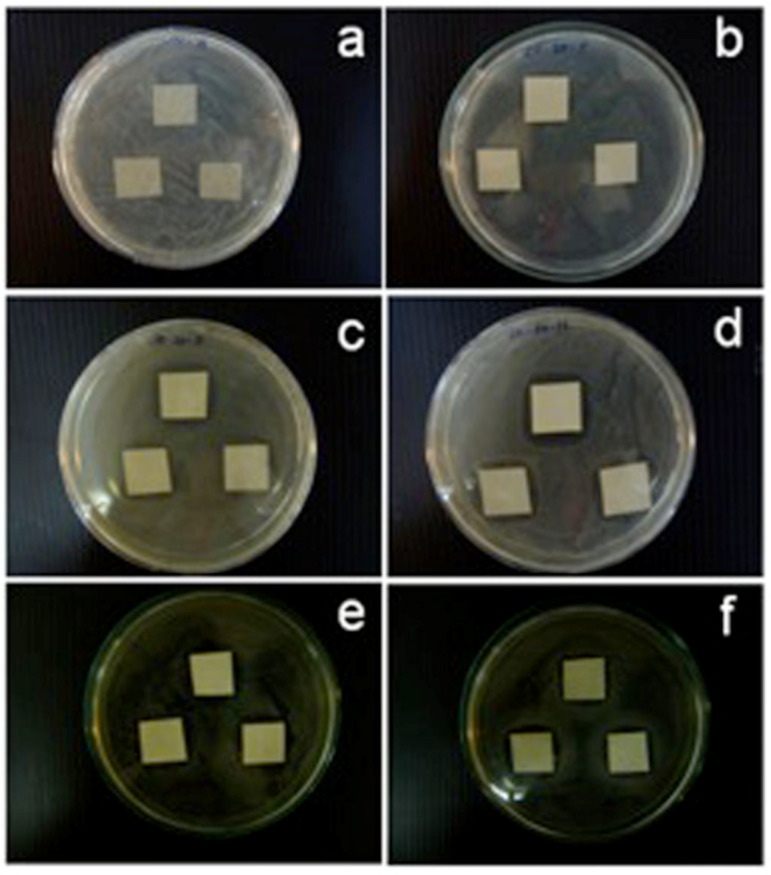
Zone of inhibition observed for different samples of antimicrobial paper under various conditions: (a) control with no ZnO after 72 h incubation of *E. coli* under halogen light; (b) 20 mM / 20 h sample after 72 h incubation of *E. coli* under fluorescent light; (c) 10 mM / 20 h sample after 72 h incubation of *E. coli* under halogen light; (d) 20 mM / 20 h sample after 72 h incubation of *E. coli* under halogen light; (e) 10 mM / 20 h sample after 72 h incubation of *S. aureus* under halogen light; (f) 20 mM / 20 h sample after 72 h incubation of *S. aureus* under halogen light.

**Figure 3:**
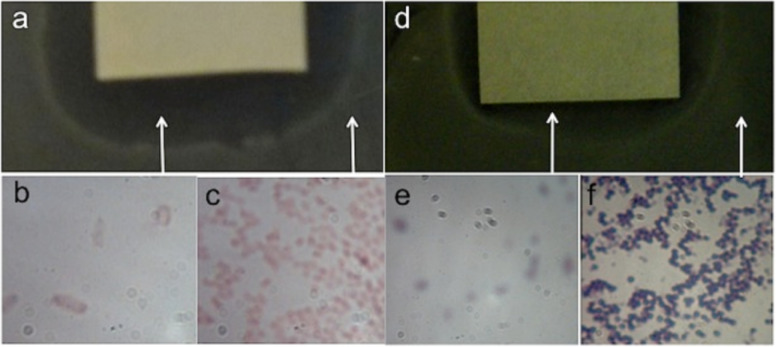
(a) Optical image of the zone of inhibition for *E. coli*; (b) *E. coli* bacterial cells in the inhibition zone; (c) *E. coli* bacterial cells outside the inhibition zone; (d) optical image of the zone of inhibition for *S. aureus*; (e) *S. aureus* bacterial cells in the inhibition zone; (f) *S. aureus* bacterial cells outside the inhibition zone.

We have observed that the antimicrobial paper not only inhibits the growth of microbes coming in contact with it but also prevents microbial growth in the area surrounding the paper [21]. The zones of inhibition around the paper samples are shown in Figure 2. In our experiments we observed comparable zones of inhibition for *E. coli* after incubation times of 24, 48 and 72 h with maximum inhibition of 2.8 cm after 72 h under halogen-light illumination. For *S. aureus,* the maximum zone of inhibition noted was 2.1 cm for 72 h incubation under 1 klx halogen light. The minimum zones of inhibition were noted for dark conditions confirming the enhancement of the inhibition process due to photocatalytic activation by the ZnO nanorods. Optical microscope images taken at 1000× within and outside of the zone of inhibition, for paper samples with ZnO nanorods grown at 20 mM for 20 h, show a negligible number of bacterial cells inside the zone of inhibition (Figure 3).

**Figure 4:**
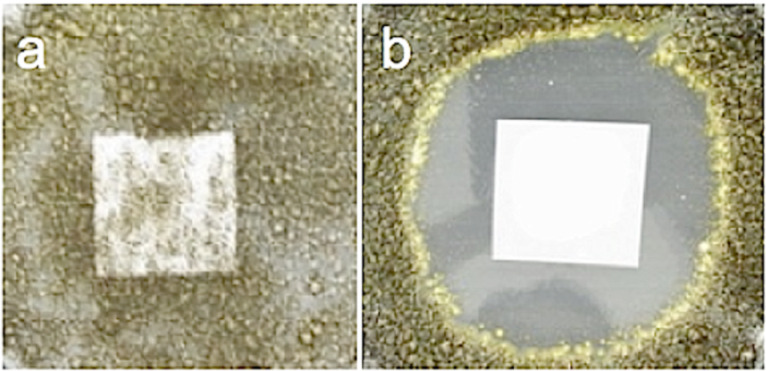
Growth of *A. niger* in the presence of (a) untreated paper and (b) paper with ZnO nanorods, after 72 h of growth.

In order to distinguish between bacteriostatic and bactericidal effects on the inhibition of bacterial growth in the area around the antimicrobial paper, bacterial cells were collected from within and outside the zone of inhibition, spread on agar plates and incubated for 48 h at 37 °C following the procedure discussed in the experimental section. No viable bacterial colonies could be found in the samples collected from the inhibition zone, whereas 7.7 × 10^8^ colonies developed after incubation from samples collected outside the inhibition zone. For *S. aureus,* the survival rate was 8 and 9.3 × 10^8^ colonies inside and outside of the zone of inhibition, respectively. These observations further confirm that the inhibition of bacterial growth is due to the bactericidal effects of the ZnO nanorods rather than bacteriostatic effects. More detailed studies using electron microscopy and fluorescence observations, which are currently in progress, will further elucidate the phenomena. The zone of inhibition in the case of *A. niger* after incubation for 72 h in the dark was measured to be 2.6 cm. The antimicrobial paper was found to inhibit the growth of fungi as well. The results from the experiments conducted with *aspergillus niger* show that a zone of inhibition almost thrice the area of the paper sample could be achieved after incubation for 72 h. The growth of *A. niger* in the presence of untreated paper and paper embedded with ZnO nanorods is shown in Figure 4a and Figure 4b, respectively, clearly demonstrating the immobilization properties of ZnO-nanorod-loaded paper samples. In Figure 4a the activity obtained was on a plain paper sample, albeit under the condition that 100 µL of the suspension of the microbial cells was spread on nutrient agar and the square paper samples.

We did not observe a marked difference in the activity with repeated seeding of microbes. This is possibly because the surface area available on the nanorods for the adsorption of the microbial cells is sufficiently high as compared to the concentration of the microbial cells. We have already reported our observations on ZnO nanorods grown on glass substrates [25].

**Figure 5:**
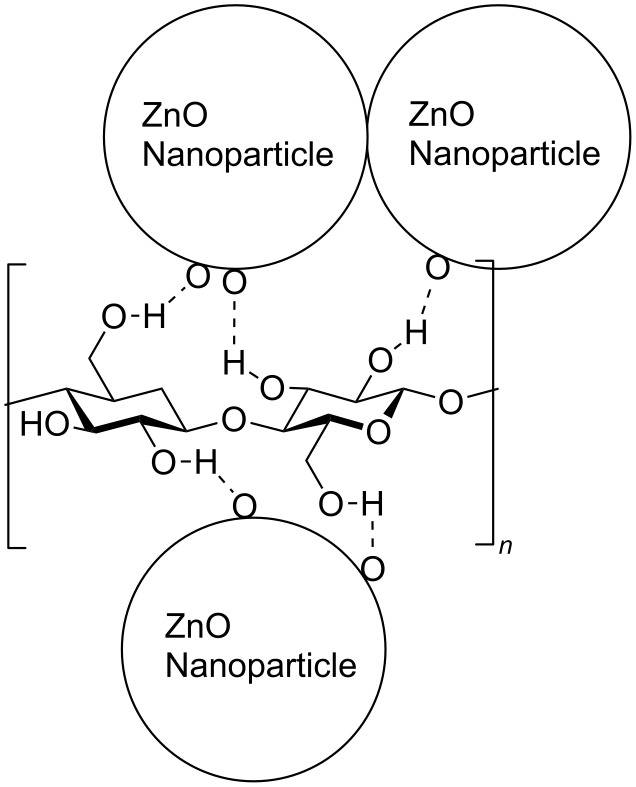
Schematic diagram showing possible hydrogen bonding of the hydroxy groups on the cellulose molecule with surface oxygen atoms of the ZnO nanoparticles.

The proper attachment of the ZnO nanorods to the paper is crucial for commercial applications. Cellulose, the major ingredient of softwood pulp, is a long-chain polymer with hydroxyl groups that can form hydrogen bonds with the surface oxygen atoms of ZnO nanoparticles. As a result, the ZnO nanoparticles that are used for nucleating the nanorods get attached to the surface without the need for any further surface treatments, such as, for example, the surface treatment of polyethylene fibers with dodecane thiol for attachment of ZnO seed nanoparticles prior to nanowire growth [26]. In Figure 5 we have schematically represented the possible hydrogen bonding of the hydroxyl ions with the oxygen atoms on the surface of the ZnO nanoparticles.

**Figure 6:**
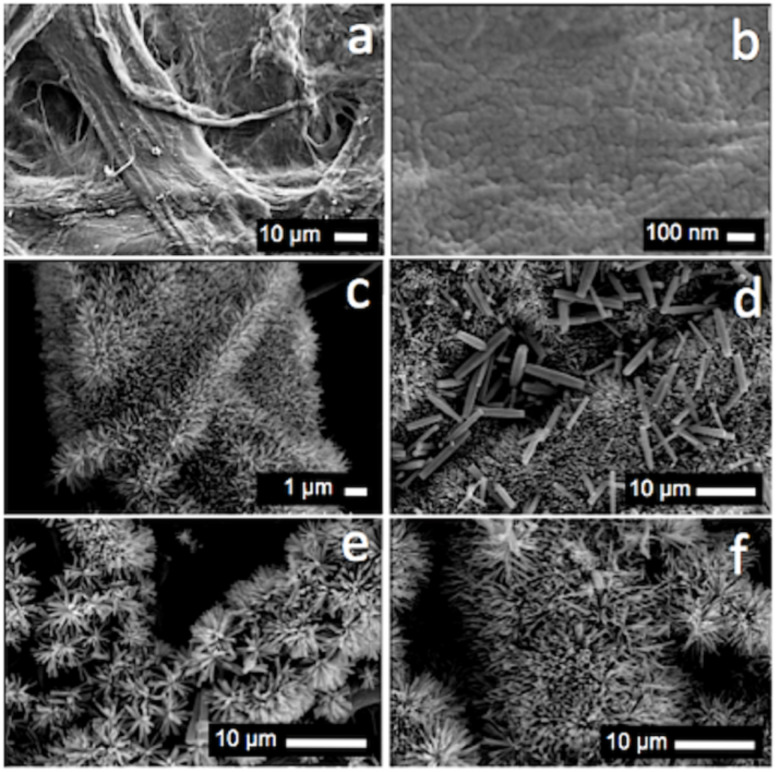
Scanning electron micrographs showing (a) untreated paper handsheet; (b) after seeding with ZnO nanoparticles; (c) ZnO nanorods grown on paper at a concentration of 10 mM for 10 h; (d) ZnO nanorods grown on paper at a concentration of 10 mM for 20 h; (e) ZnO nanorods grown on paper at a concentration of 20 mM for 10 h; and (f) ZnO nanorods grown on paper at a concentration of 20 mM for 20 h.

In order to test the attachment of the nanorods on the paper substrate, air was allowed to pass through the paper at a pressure of 2 bar. It was observed that after an initial weight loss in the first 2 min due to the removal of loosely attached ZnO agglomerates, the weight of the paper was constant even after continuous blowing of air for 10 min [21]. All SEM images shown in this manuscript were recorded on samples that had been cleaned with pressurized air, demonstrating that indeed ZnO nanorods do stay attached to the antimicrobial paper developed during this study. SEM micrographs of the paper substrate before and after seeding with ZnO nanoparticles are shown in Figure 6a and Figure 6b. Figure 6c and Figure 6d show ZnO nanorods grown on paper by using a 10 mM zinc nitrate and hexamine growth solution for 10 h and 20 h, respectively. The ZnO nanorods grown with a 20 mM concentration of reactants (Figure 6e and Figure 6f) are thicker and longer as compared to the ones grown at 10 mM [27–28].

The dimensions of the ZnO nanorods grown under different conditions are given in Table 1. The overall loading of ZnO on the paper matrix is about 200 μg/cm^2^ for the sample onto which the nanorods were grown in a reaction bath with a concentration of 10 mM of zinc nitrate and hexamethylenetetramine, and about 1.2 mg/cm^2^ for the sample grown at a concentration of 20 mM.

**Table 1:** Widths and lengths of ZnO nanorods grown at different concentrations of zinc nitrate and hexamine for different durations (50 nanorods sampled for each).

Conc. of zinc nitrate and hexamine (mM)/synthesis time (h)	Width (nm)	Length (nm)

10/10	60–100	500–600
10/20	100–150	800–1,000
20/10	250–300	1,800–2,200
20/20	250–350	3,400–4,200

**Figure 7:**
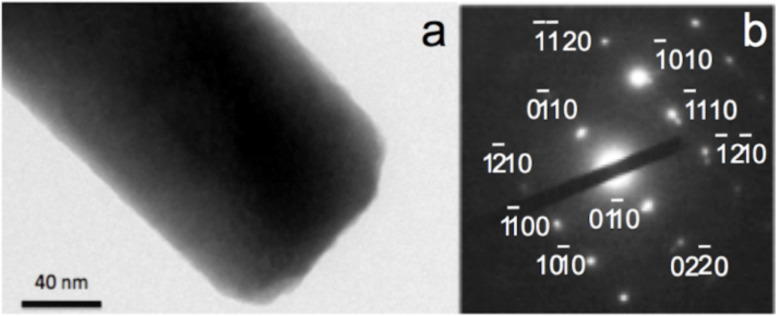
(a) Transmission electron microscopy (TEM) image of a single ZnO nanorod (b) Electron diffraction pattern showing the single crystalline structure. The planes from which the electron beam was diffracted to generate the diffraction pattern are indexed in the diffractogram.

The ZnO nanorods grown on the paper supports are single crystalline, which was confirmed from the electron diffraction pattern shown in Figure 7b. The diffraction pattern was taken on the ZnO nanorod shown in the TEM micrograph in Figure 7a. The indexed planes confirm the wurtzite structure of ZnO (Figure 7b).

The addition of functionalities to paper should not have a detrimental effect on its inherent properties, such as brightness, ink-retention capability, etc. Addition of Ag nanoparticles gives a yellowish tinge to the paper thereby affecting its brightness. For the paper reported here, the brightness increased upon inclusion of the ZnO nanorods, which is expected as ZnO and TiO_2_ particles scatter light and are used for paper-brightening processes [29]. Results from the brightness test are tabulated in Table 2. The brightness was found to increase by ~4% for all the samples compared to the untreated paper, which is attributed to the scattering of light by the ZnO nanorods [30–31].

**Table 2:** Brightness of the paper samples with and without ZnO nanorods measured by using a Technidyne Color Touch PC.

Concentration of reactants in growth bath (mM)	Growth duration (h)	Brightness (%)

Treated samples		

10	10	81.32
10	20	80.90
20	10	81.13
20	20	80.44

Untreated paper		

–	–	76.74

**Figure 8:**
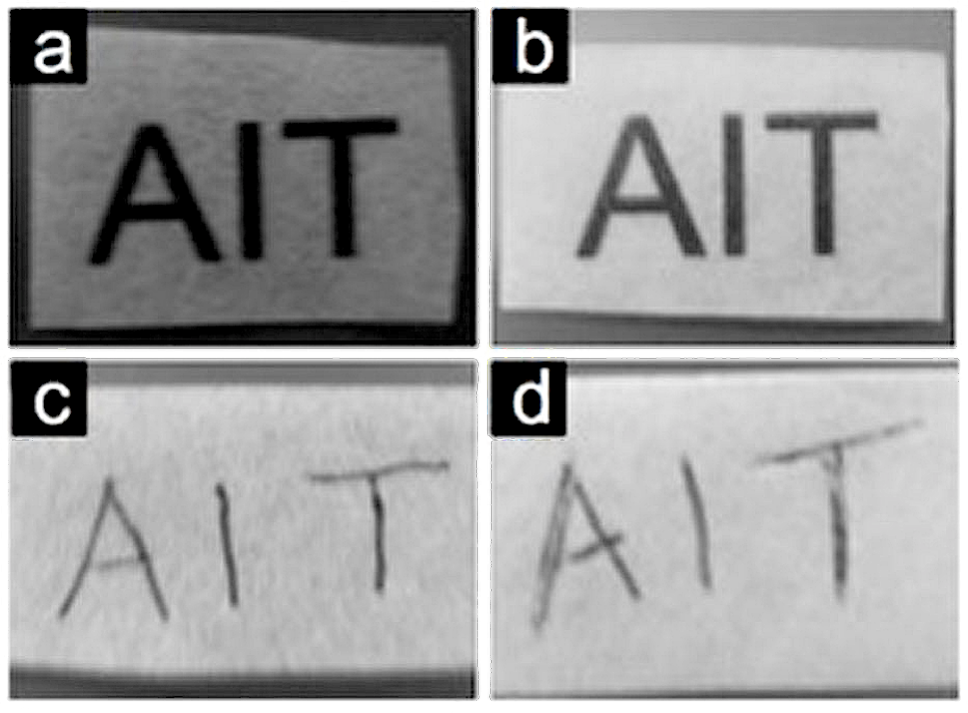
(a) Ink-jet printed text on untreated paper and (b) ink-jet printed text on paper coated with ZnO nanorods (20 mM / 20 h). (c) Text written with a ball-point pen on untreated paper and (d) text written with ball-point pen on paper coated with ZnO nanorods (20 mM / 20 h).

The antimicrobial paper incorporated with ZnO nanorods reported here could be used for writing as well as printing with the same quality as the untreated paper. Figure 8 shows digital images of the untreated paper and a sample of the antimicrobial paper grown at a concentration of 20 mM for 20 h with hand-written and printed text. The antimicrobial paper can therefore be used for various healthcare applications such as face masks, writing and printing paper, and tissue papers.

## Conclusion

Antimicrobial paper has been successfully prepared by growing ZnO nanorods on paper prepared from bleached soft wood kraft pulp by using a simple hydrothermal process at low temperature. The antimicrobial paper inhibits the growth of harmful microbes due to a slow release of zinc ions and the inhibition is further enhanced through photocatalysis under room lighting conditions. The antimicrobial paper was successfully used to immobilize two common bacteria, *E. coli* and *S. aureus*, in the immediate vicinity of the paper. The photocatalytic effect of the paper containing ZnO nanorods on the Gram-negative bacterium *E. coli* is more pronounced, with inhibition zones of 2.8 cm under halogen lighting and 2.4 cm under fluorescent lighting around the square samples of edge 1.5 cm after 72 h of incubation. The growth of the Gram-positive bacterium *S. aureus* could be inhibited in a zone of 2.1 cm under halogen lighting and 1.9 cm under fluorescent lighting. The antimicrobial paper was also observed to inhibit the growth of the fungus *A. niger* with an inhibition zone of 2.6 cm. Antimicrobial papers based on ZnO nanorods can find wide applications as wallpaper, cleaning tissue, writing paper and as a facemask material.

## Experimental

Circular sheets of paper of 15.9 cm diameter were prepared following a process explained in detail in a previous work [21]. The raw material used was bleached soft wood kraft pulp, which was refined by using a port fuel injection (PFI) mill to form paper handsheets with a base weight of 35 g/m^2^. The paper sheets were at first seeded with ZnO nanoparticles by dip coating (three times) and the samples were subsequently dried in an oven maintained at 90 °C for 15 min after every successive dipping.

The ZnO nanoparticles used for seeding were synthesized in an ethanolic colloidal solution following a procedure reported previously [32–33]. In short, 20 mL of 4 mM zinc acetate dihydrate [(CH_3_COO)_2_ Zn·2H_2_O, Merck] solution was mixed with 20 mL of fresh ethanol and heated at 70 °C for half an hour. The solution was then cooled to room temperature and 20 mL of 4 mM sodium hydroxide [NaOH, Merck] solution was then added. The admixture was subsequently hydrolyzed at 60 °C for 2 h, which resulted in a transparent colloidal dispersion of ZnO nanoparticles.

ZnO nanorods were grown following a low-temperature hydrothermal growth process [34–35]. The seeded paper substrates were dipped in an equimolar solution of zinc nitrate hexahydrate [Zn (NO_3_)_2_·6H_2_O, APS Ajax Finechem] and hexamethylenetetramine [(CH_2_)_6_N_4_, Carlo Erba], and the admixture was maintained at 90 °C for up to 20 h, which led to the growth of hexagonal ZnO nanorods. Two different concentrations of zinc nitrate and hexamine were used for the nanorod growth in this work, i.e., 10 mM and 20 mM, with growth durations varied between 5, 10 and 20 h in both the cases. The reaction bath was replenished every five hours to maintain the growth rate, as discussed elsewhere [26]. The substrate was then removed and washed with deionized water several times and then dried at 70 °C for 6 h.

Scanning Electron Microscopy (SEM) was carried out on a JEOL JSM-6301F SEM at an operating voltage of 20 kV. Quantitative measurements on SEM micrographs were carried out by using Scion image-processing software. For transmission Electron Microscopy (TEM), a JEOL/JEM 2010 operated at 120 kV was used. Samples for TEM were prepared by scraping off ZnO nanorods from a glass substrate grown under similar conditions. A diluted aqueous suspension of the ZnO nanorods was carefully dropped on a copper-coated TEM grid and dried in air. Brightness of the paper was measured by using a Technidyne Color Touch PC from IDM Instruments.

Antimicrobial tests were carried out following a protocol already reported in a previous publication [21]. *E.coli* (strain TISTR 073) and *S. aureus* (strain KU) cells, procured from the Thailand Institute of Scientific and Technological Research and Kasetsert University, Thailand, respectively, were cultivated on nutrient agar (Difco, USA) and incubated at 37 °C for 18 h following the streak-plate method [36]. Following incubation for 18 h, cells were scraped from the nutrient agar, and mixed with phosphate buffer saline (PBS) solution (8 g NaCl, 0.2 g KCl, 1.44 g Na_2_HPO_4_, 0.24 g KH_2_PO_4_ in 1 L of distilled water) by a vortex mixer until the cells were homogeneously dispersed. The suspension was centrifuged at 4,000 rpm for 10 min and the supernatant discarded. These steps were repeated twice, and the cell pellet was mixed with 1 mL of deionized water for the experiments. The *E. coli* cells thus prepared were redispersed in 10 mL of Milli-Q water, and the optical density (OD) of the suspension was measured by using a UV–vis spectrophotometer at a wavelength of 600 nm. *A. niger* was cultivated for 72 h at room temperature on potato dextrose agar (PDA) medium. The fungal conidia was then mixed with 10 mL of sterile water by using a vortex mixer for 1 min to break up conidia chains and separate conidia from mycelia [37]. The conidia suspension was taken on a microscope to count the number of conidia by using a counting chamber. The conidia suspension was adjusted to 9 × 10^4^ conidia/mL by adding sterile deionized water.

To test the antimicrobial efficacy of the paper, three square samples (1.5 cm × 1.5 cm) were cut from both the untreated and ZnO treated papers from three different batches, as described in [21]. The samples were dried in a laminar air flow for 10 min prior to conducting antimicrobial tests. The antimicrobial activity was observed considering the zone of inhibition (absence of viable microbial cells) around the paper samples. For the zone-of-inhibition test, 100 µL of the suspension of the microbial cells was spread on nutrient agar and the square paper samples were placed on it in a triangular formation. The temperature inside the incubation box was maintained at 37 °C. After incubation for a total duration of 72 h, the area of inhibition (absence of viable cells) was measured and optical images taken. *E. coli* and *S. aureus* were stained with safranin and crystal violet, respectively, before the optical images were taken. The antibacterial tests were carried out by using different samples of the antimicrobial paper under three different illumination conditions, i.e., in the dark, under light from a fluorescent lamp (1 klx), or under light from a tungsten–halogen lamp (1.2 klx). The intensity of light was kept comparable to standard room lighting conditions.
